# Proteomic Analysis of the Cell Cycle of Procylic Form Trypanosoma brucei*[Fn FN2]

**DOI:** 10.1074/mcp.RA118.000650

**Published:** 2018-03-19

**Authors:** Thomas W. M. Crozier, Michele Tinti, Richard J. Wheeler, Tony Ly, Michael A. J. Ferguson, Angus I. Lamond

**Affiliations:** From the ‡Wellcome Centre for Anti-Infectives Research, School of Life Sciences, University of Dundee, Dundee, DD1 5EH, UK;; §Centre for Gene Regulation and Expression, School of Life Sciences, University of Dundee, Dundee, DD1 5EH, UK;; ‖Sir William Dunn School of Pathology, University of Oxford, Oxford, OX1 3RE, UK

**Keywords:** Cell cycle*, Cell division*, Gene Expression*, Infectious disease, Mass Spectrometry, Mitosis, Molecular biology*, Cell biology*, Parasite, Quantification, procyclic, Trypanosoma

## Abstract

We describe a single-step centrifugal elutriation method to produce synchronous Gap1 (G1)-phase procyclic trypanosomes at a scale amenable for proteomic analysis of the cell cycle. Using ten-plex tandem mass tag (TMT) labeling and mass spectrometry (MS)-based proteomics technology, the expression levels of 5325 proteins were quantified across the cell cycle in this parasite. Of these, 384 proteins were classified as cell-cycle regulated and subdivided into nine clusters with distinct temporal regulation. These groups included many known cell cycle regulators in trypanosomes, which validates the approach. In addition, we identify 40 novel cell cycle regulated proteins that are essential for trypanosome survival and thus represent potential future drug targets for the prevention of trypanosomiasis. Through cross-comparison to the TrypTag endogenous tagging microscopy database, we were able to validate the cell-cycle regulated patterns of expression for many of the proteins of unknown function detected in our proteomic analysis. A convenient interface to access and interrogate these data is also presented, providing a useful resource for the scientific community. Data are available via ProteomeXchange with identifier PXD008741 (https://www.ebi.ac.uk/pride/archive/).

The eukaryotic mitotic cell division cycle is an evolutionarily conserved process in which a cell duplicates and segregates newly synthesized cellular components to produce two progeny cells from a single mother cell. The synthesis and degradation and/or activation and inactivation of regulatory proteins controls the temporal order of events that must occur for cell division to proceed correctly. The cell division cycle can be separated into four consecutive phases: Gap1 (G1), DNA synthesis (S), Gap2 (G2), and mitotic (M) phases. The key events of cell division include DNA replication (S phase) and segregation of replicated DNA (M-phase), interceded by the two “gap” phases, G1- and G2-phase, where cells either sense environmental conditions prior to commitment to cell division, or assess completion of DNA replication prior to entry into mitosis, respectively. These events must occur in order and only once per mitotic cell division (1).

*Trypanosoma brucei* is an evolutionarily divergent eukaryotic protozoan parasite that causes human and animal trypanosomiasis in sub-Saharan Africa. Current therapeutics for these diseases suffer from issues of toxicity and complexity of administration. Genomic sequencing of *T. brucei* in 2005 identified ∼9100 genes, ∼4900 of which encode predicted proteins that lack reliable orthologues in other organisms and are annotated as “hypothetical,” hampering our understanding of trypanosome biology and associated therapeutic possibilities. At the time of writing, ∼3000 out of 8324 orthologous genes are annotated as hypothetical proteins.

*T. brucei* shares much of its basic cell cycle regulatory machinery with other eukaryotes. For example, the *T. brucei* genome contains multiple cyclins and Cdc2-related kinases (CRKs)^1^, different pairs of which are necessary for transitions between the G1/S and G2/M-phases of the cell cycle (2–5). On the other hand, components thought to be essential for cell division in other eukaryotes, such as the spindle assembly checkpoint, have so far not been identified in trypanosomatid species (6–8), whereas trypanosome kinetochore orthologues have only been recently discovered (9). Further, trypanosomes contain unique single-copy organelles such as the basal body, the flagellum, the mitochondrion and the kinetoplast (mitochondrial DNA network) that must be duplicated and segregated equally to produce viable progeny cells. The molecular machineries controlling this highly regulated coordination of organelle duplication and segregation are not well understood.

Previous transcriptomic analyses of the cell cycle in *T. brucei* uncovered novel components of cell division unique to trypanosomatids, and thus identified attractive potential drug targets (9, 10). However, it is acknowledged that, in an organism that controls gene expression post-transcriptionally through RNA binding proteins, the transcriptome is not a perfect proxy for the proteome (11–14). The proteomic analysis described here is designed to complement previously published transcriptomic data and further contribute to our understanding of cell cycle control in trypanosomes (10). To this end, we have adapted methods for producing populations of synchronous G1-phase procyclic form (PCF) *T. brucei* at a scale amenable for multi time point proteomic analyses, without the use of chemical agents to synchronize the cells.

Centrifugal elutriation has been used for the cell-cycle synchronization of procyclic and bloodstream form trypanosomes previously (15). Using 10-plex Tandem Mass Tag (TMT) labeling, in conjunction with mass spectrometry (MS)-based proteomics technology, we quantified the relative abundance of 5325 proteins in PCF *T. brucei* across nine time-points of cell division, for three biological replicates. We identified many known cell cycle regulated proteins, thereby validating our approach. We also identified cell cycle regulated patterns of expression for 151 “hypothetical proteins of unknown function,” 40 of which are thought to be essential for parasite survival in culture and may, therefore, be interesting future candidates as drug targets. Finally, through cross-comparison to the TrypTag microscopy database (16), we validate the cell cycle regulated patterns of expression for many hypothetical proteins of unknown function.

## EXPERIMENTAL PROCEDURES

### 

#### 

##### SDM-79 Media Preparation

Powdered SDM-79 media was dissolved in water and supplemented with hemein to 7.5 mg/L and 2 g/L of sodium bicarbonate. The pH was adjusted to 7.3 with NaOH, and sterile filtered using Stericups 500. Under sterile conditions, heat inactivated and non-dialyzed fetal bovine serum (PAA) was added to 15% (v/v) and Glutamax I to 2 mm. The antibiotics, G418 and hygromycin, were used at final concentrations of 15 μg/ml and 50 μg/ml respectively.

##### Cell Culture

Procyclic trypanosomes (clone 29.13.6) were cultured in SDM-79 media at 28 °C, without CO_2_, in fully capped culture flasks.

##### Direct Elutriation

Procyclic cells (2.7 × 10^9^) were harvested from 100 ml of a log-phase culture by centrifugation and resuspended in 10 ml of elutriation buffer (a 1:4 dilution of SDM-79 in PBS). Cells were passed twice through a 20-gauge needle to disperse any cell aggregates and injected into a Sanderson loading chamber of an Avanti J-26 XP elutriation centrifuge equipped with JE5.0 rotor at a temperature of 28 °C. Cells were loaded at a flow rate of 10 ml/min. The rotor was kept at a constant speed of 5,000 rpm. Fractions of 50 ml were collected at each flow rate of 10, 15, 18, 20, 23, 25, 27, 28, 29, 31, 32, 33 and 35 ml/min. The final fraction was collected at 35 ml/min with the rotor turned off. Aliquots were taken from each collected fraction for flow cytometry analysis.

##### Single and Double-cut Elutriation

Cells were prepared in a similar manner as described for *Direct Elutriation*. Cells were collected at two flow rates—15 ml/min (small cells) and 32 ml/min (large cells). It has been noted that at the same centrifugal speed (5000 rpm), higher flow rates (18–22 ml/min) can be used to separate cells in distinct temporal stages of G1 (15). Both collected cell populations were pelleted by centrifugation, resuspended in SDM-79 at a concentration of 3 × 10^7^ cells/ml and placed in culture. Aliquots of the “single-cut” small cell culture were taken for flow cytometry at 0.5, 3, 4, 5, 6, 7, 8, 9, 10, and 11 h after elutriation. The large cell population was cultured for 1 h and re-elutriated, collecting and placing into culture only the newly-divided small cells. Aliquots of this “double-cut” culture were also taken for cytometry at the postelutriation time intervals.

##### Flow Cytometry

Cells (1 × 10^6^) were washed three times in 5 ml PBS, fixed in 1 ml 70% ice-cold ethanol and stored at −20 °C before DNA staining for flow cytometry. Fixed cells were washed with 1 ml of PBS and resuspended in staining solution composed of 50 μg/ml propidium iodide, 100 μg/ml ribonuclease A, 0.5% (w/v) Triton-X100 and 0.5% bovine serum albumin in PBS. Cells were incubated in the dark at room temperature for a minimum of 20 min. Propidium iodide fluorescence was detected from 10,000 cells per sample on an LSR Fortessa cytometer.

##### TMT Labeling of Samples from Single-cut Elutriation

**Fig. 1.**
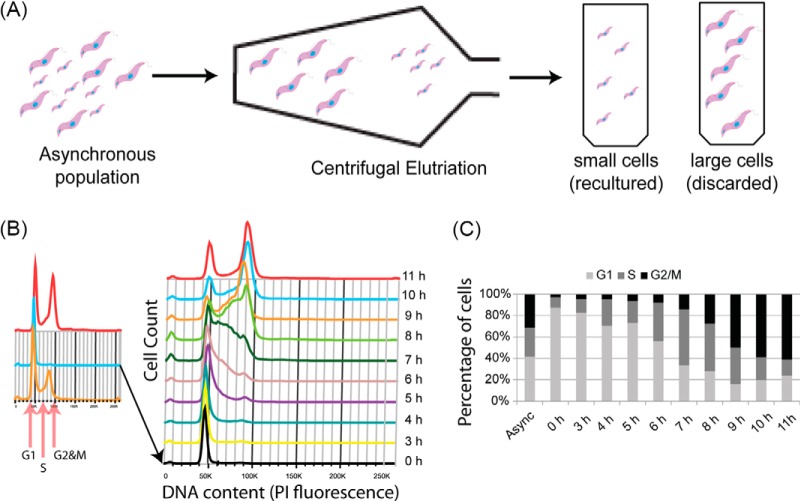
**Single Cut Elutriation.** *A*, Diagrammatic representation of single-cut elutriation. An asynchronous culture of procyclic form trypanosomes is loaded into the elutriation chamber and split into “small” cells, which are re-cultured, and “large” cells, which are discarded. *B*, DNA content (PI staining) of cells harvested during single-cut elutriation. Inset panel shows asynchronous, small and large cells. Time-course represents cells harvested from the re-cultured “small” cell population. *C*, Estimation of cell cycle distribution of cells harvested from time-course compared with original asynchronous culture.

Three biological replicates of single-cut elutriation were performed, and cultures were subsequently seeded with small (G1) cells at 3 × 10^7^ cells/ml. Samples of ∼1.5 × 10^8^ cells were harvested 0.5, 3, 5, 6, 7, 8, 9, 10 and 11 h after the initiation of the cell cultures (Fig. 1). At each time point cells were washed in PBS at 4 °C prior to lysis in 200 μl of 4% SDS, 10 mm sodium phosphate (pH 6.0), 100 mm NaCl, 25 mm Tris(2-carboxyethyl)phosphine hydrochloride and 50 mm N-ethylmaleimide (NEM). Lysates were sonicated in a Bioruptor Pico (Diagenode) water bath sonicator for 10 min, then heated to 65 °C for 10 min before chloroform-methanol precipitation.

For chloroform-methanol precipitation, one volume of lysate (200 μl) was mixed with four volumes of methanol, one volume of chloroform and three volumes of water and vortexed for 1 min. Samples were centrifuged at 9000 × *g* for 5 min at room temperature in a bench-top centrifuge. The upper phase was removed, carefully avoiding the interface of precipitated protein. Three volumes of methanol were added, and the sample centrifuged again, followed by removal of all remaining supernatant. Protein pellets were air-dried and resuspended in one volume of 8 m urea, 1 mm CaCl_2_ in 0.1 m Tris-HCl (pH 8.0).

Protein concentrations were determined by Bradford assay for each time point and LysC added at a 1:100 ratio of protein to protease and digested overnight at 37 °C. Samples were diluted to 1 m urea with 0.1 m Tris-HCl (pH 8.0) and 1 mm CaCl_2_ and trypsin added at the same ratio. Digestion proceeded for 6 h prior to acidification of samples with trifluoroacetic acid (TFA) to 1%. Each time point was separately loaded onto a 500 mg SepPak cartridge (Waters) that had been wetted with 100% acetonitrile and equilibrated with 0.1% aqueous TFA. Adsorbed peptides were washed with 4 ml 0.1% TFA, eluted in 1 ml of 50% acetonitrile and 0.1% TFA, dried using a GeneVac evaporator and resuspended in 50 mm HEPES (pH 8.5) with 123 μg of peptide, as determined using a CBQCA reagent assay (Thermo), from each sample used for TMT labeling.

**Fig. 2.**
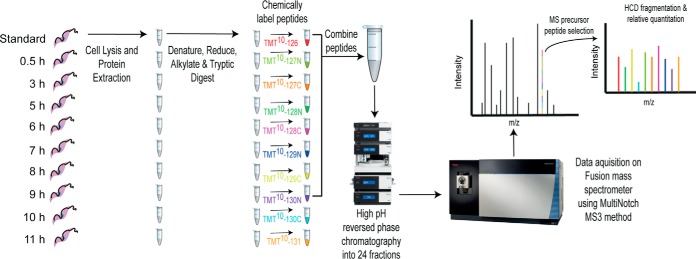
**Diagrammatic representation of workflow for protein quantitation.** Cells from each harvested time point were lysed, and extracted proteins were processed to produce reduced, alkylated tryptic peptides separately. Peptides were chemically labeled with the indicated tandem-mass tags and were combined at a 1:1 ratio following quenching of the labeling reaction. The combined peptides were fractionated by high-pH reverse phase chromatography into 24 fractions which were prepared for mass spectrometry and acquired on a Fusion mass spectrometer using the MultiNotch MS3 method (47).

TMT ten-plex reagents (ThermoFisher) were used to label the samples from each biological replicate (Fig. 2). Aliquots (0.8 mg) of each reagent in 41 μl of anhydrous acetonitrile were incubated with peptide samples for 2 h at room temperature. The reaction was quenched by the addition of 8 μl of 5% hydroxylamine followed by incubation for 15 min at room temperature. Nine of the ten TMT reagents were used to label the nine time-points collected in each biological replicate, and one was used to label a reference peptide sample, made by mixing together equal aliquots of peptide from each time point. For each biological replicate, equal amounts of the ten TMT-labeled samples (nine time-points and one reference) were mixed and the TMT-labeled peptides were purified on a SepPak cartridge, as described above. The resulting dried TMT-labeled peptides were solubilized in 2% acetonitrile in 10 mm ammonium formate (pH 9.0) for high-pH reverse phase chromatography.

##### High-pH Reverse Phase Chromatography

TMT labeled peptides were injected onto an Xbridge BEH C18 column (130 Å, 3.5 μm, 4.6 × 150 mm), using a Dionex Ultimate 3000 HPLC system. Buffer A was composed of 2% acetonitrile in 10 mm ammonium formate (pH 9.0) and buffer B of 80% acetonitrile in 10 mm ammonium formate (pH 9.0). Columns were run at 1 ml/min at 30 °C, starting at 35% buffer B, and rising to 60% B over the course of a 0–11 min linear gradient. Buffer B was increased to 100% from 11 to 12 min followed by a drop back to 35% B from 12 to 13 min and this was maintained until the end of the run at 20 min. Fractions were collected from 2 to 16 min with 8.75 s per fraction, producing 96 fractions. Fractions were collected into 24 samples, for example the 1st, 25th, 49th and 73rd fractions were pooled in the same well of a 96-well plate. The 24 samples per biological replicate were dried using a GeneVac evaporator and solubilized in 5% formic acid.

##### LC-MultiNotch-MS3 and Analysis of Spectra

A total of 1 μg of peptide for each of the 24 samples was injected onto a C18 nano-trap using a Thermo Scientific Ultimate 3000 nanoHPLC system. Peptides were washed with 2% acetonitrile, 0.1% formic acid and separated on a 150 mm × 75 μm C18 reverse phase analytical column with a 120 min, 2% to 28% acetonitrile gradient at a flow rate of 200 nL/min. Peptides were ionized by nano-electrospray ionization at 2.5 kV. Data was acquired for each sample in triplicate.

Survey scans were performed with a Thermo Fisher Fusion mass spectrometer, using the Orbitrap at a resolution of 120,000 over a range of 350–1400 *m*/*z* with an AGC target of 2 × 10^5^ and a maxIT of 300 ms. Monoisotopic ion precursor selection was turned on, and only ions with a charge state between 2–7 and a minimum intensity of 5 × 10^3^ were selected for fragmentation. Ions selected were excluded from further selection for 40 s. A 1.6 *m*/*z* isolation width was used to select ions from the MS1 survey scan for Collision Induced Dissociation fragmentation at a normalized collision energy of 30%. Scans of fragment ions were acquired using the ion trap in Rapid Scan mode with an AGC target of 1 × 10^4^ and a 70 ms maxIT. Fragment ions were selected for further fragmentation using Synchronous Precursor Selection. Fragment ions were selected from 400–1200 *m*/*z* and excluded ions 20 *m*/*z* below or 5 *m*/*z* above the precursor ion mass, and *m*/*z* ratios correlating to the loss of TMT from the precursor ion. The top 10 most intense fragment ions were selected for HCD fragmentation with a 55% normalized collision energy and an isolation width of 2 *m*/*z*. MS3 scans were acquired using the Orbitrap at a resolution of 60,000 from 100–500 *m*/*z*, an AGC target of 1 × 10^5^ and a maxIT of 150 ms. The cycle time between MS1 survey scans was set to 2 s.

RAW data files were analyzed using MaxQuant version 1.5.3.8, with the in-built Andromeda search engine (17, 18), supplied with the *T. brucei brucei* 927 annotated protein database from TriTrypDB release 26.0 containing, 11,567 entries. The mass tolerance was set to 4.5 ppm for precursor ions and MS/MS mass tolerance was set at 20 ppm. The enzyme was set to trypsin and endopeptidase LysC, allowing up to 2 missed cleavages. NEM on cysteine was set as a fixed modification. Acetylation of protein N termini, deamidation of asparagine and glutamine, pyro-glutamate (with N-terminal glutamine), oxidation of methionine and phosphorylation of serine, threonine and tyrosine were set as variable modifications. The false-discovery rate for protein and peptide level identifications was set at 1%, using a target-decoy based strategy. Only unique peptides were used for quantitation. The results can be viewed from the MS-Viewer website (19) by entering the search key, t5jurduitz.

##### Experimental Design and Statistical Rationale

Centrifugal elutriation experiments were repeated in triplicate to produce three biological replicates for analysis. Each biological replicate was fractionated into 24 fractions, separated by high-pH reverse phase chromatography, each of which was run in technical triplicate. Proteins were classified as cell cycle regulated if they were detected in a minimum of two biological replicates, with a Pearson correlation > 0.7 and a mean fold change > 1.3. Proteins identified with one unique peptide were included in this analysis because of the stringent Pearson correlation cut-off, ensuring data from these peptides were highly reproducible.

##### Data Analysis

The three biological replicates were normalized with a recently described technique named CONSTANd (20). Briefly, this method adopts an iterative proportional fitting procedure to constrain the row means and column means to be equal to the constant (C) value of one divided by the number of TMT quantitation channels. This constraint is achieved by a series of iteration steps. Each iteration step is composed of two phases. In the first phase, the row values are divided by the row mean and multiplied by the number of channels. In the second phase, the column values are divided by the column mean and multiplied by the number of channels. The iterative process repeats until either the rows L1 error, or the columns L1 error, is less than 1e^−5^. The L1 error is defined as the sum of the absolute differences between the row or column averages and the C value.

After normalization, the mean time point values and the Pearson correlation coefficient (PCC) of the three experimental replicates were computed for each protein detected with ≥ 1 unique peptide. The maximum fold-change (MFC) was calculated by dividing the maximum detected time point by the minimum detected time point for each protein. Proteins were classified as cell cycle regulated if they were detected in at least two out of three biological replicates; had a PCC or mean PCC greater than 0.7 if detected in either two, or three experiments, respectively, with a MFC greater than 1.3.

Proteins were clustered into nine groups with the Python scikit-learn package using the K-means algorithm (21). The clustering algorithm was trained with a stringent selection of the cell cycle-regulated proteins, identified in all three biological replicates, with an average PCC greater than 0.8, and a fold change greater than 1.5 (99 out of 384 proteins). The trained algorithm was applied to all the cell cycle regulated data set. The optimal number of clusters was derived with the fuzzy partition coefficient score.

The gene ontology (GO) term enrichment analysis was performed with the goatool python package (https://github.com/tanghaibao/goatools). The GO term annotation file (go-basic.obo) was downloaded from http://geneontology.org/ontology/go-basic.obo on the 10/06/17. The GO term associations file with the *T. brucei* gene IDs was compiled by parsing the gene search output from the TryTripDB database (22). For the GO term analysis, all proteins identified in the three biological replicates were used as background for the computation of the *p* value. The essential genes were retrieved from a recently published phenotype screening (23). The cell cycle regulated mRNAs classified with the appropriate phase (early or late G1, S, or G2 and M phase) according to Archer *et al.* (10). The TryTripDB database was used to retrieve the proteins annotated with GO terms associated to the cell cycle (GO:0000281: mitotic cytokinesis, GO:0051726: regulation of cell cycle, GO:0007052: mitotic spindle organization, GO:0007088: regulation of mitotic nuclear division, GO:0007067: mitotic nuclear division, GO:0051726: regulation of cell cycle, GO:0000278: mitotic cell cycle, GO:0007076: mitotic chromosome condensation, GO:0000070: mitotic sister chromatid segregation, GO:0051228: mitotic spindle disassembly, GO:0051225: spindle assembly, GO:2000134: negative regulation of G1/S transition of mitotic cell cycle, GO:0010389: regulation of G2/M transition of mitotic cell cycle).

##### TrypTag

Images from TrypTag were kindly sourced *via* Richard Wheeler from the TrypTag database (16).

## RESULTS

### 

#### 

##### Counterflow Centrifugal Elutriation

“Direct” counterflow centrifugal elutriation was used to attempt to enrich for cells in either G1, S, or G2 and M phases of the cell cycle. Fractions were collected by gradually increasing counterflow rates and were analyzed by flow cytometry to determine the cell cycle distribution of collected populations (supplemental Fig. S1). The maximum enrichment in any fraction collected for G1, S or G2 and M-phase cells was 93%, 34 and 52% respectively (supplemental Table S1). Because the enrichment of S and G2 and M-phase cells were relatively low, we chose instead to inoculate cultures with a G1-phase enriched population of cells and harvest cells at various time-points after inoculation to obtain S and G2 and M-phase cells. Two methods were compared for the intended aim of producing synchronous G1-phase enriched cell populations. Single-cut elutriation splits an asynchronous culture into “large” and “small” cells. The small cells, which are enriched in G1-phase, were used for culture inoculation (Fig. 1). Double-cut elutriation (10) involves taking the large cell population from a first elutriation and culturing them for 1–2 h before a second round of elutriation, where small, newly divided cells, are taken as the G1-phase enriched cell population (supplemental Fig. S2). In both cases, aliquots were taken over an 11 h time-course for flow cytometry analysis.

The maximum enrichment for G1, S, and G2 and M-phase cells was 88%, 53% and 61% using the single-cut method and 83%, 63%, and 68% using the double-cut method (supplemental Table S1). Although the enrichment for G1-phase cells was similar, the single-cut elutriation yields significantly more cells compared with double-cut (20% and 5% of the original cell number, respectively). Therefore, single-cut enrichment was utilized for all further studies.

##### Cell Cycle Regulated Proteome

From three biological replicates, with LC-MS/MS data acquired using three technical replicates, a total of 45,195 peptide sequences were identified corresponding to 6591 protein groups, with 5325 detected and quantified across all nine time-points in at least two biological replicates with ≥1 unique peptide. The relative quantification of peptides and proteins are derived from the intensities of ten isobaric reporter Tandem Mass Tags, which are low molecular weight tags used to label peptides from each collected time point separately, prior to pooling into one sample per biological replicate. Fragmentation of peptides releases reporter fragment ions that are observed as ten distinct low *m*/*z* ions, the relative intensities of which indicate the relative abundance of the fragmented peptide from each of the ten time-points (Fig. 2). For visualization purposes, protein abundances were normalized by setting the maximum reporter intensity per protein to 1.

Proteins defined as cell cycle regulated were required to be detected in a minimum of two out of three biological replicates (mean 2.9 replicates) with ≥ 1 unique peptide (mean 4.8 unique peptides), with mean Pearson correlation coefficients between biological replicates ≥ 0.7 and a maximum fold change ≥ 1.3 (mean fold change 1.7). According to these criteria, 384 proteins were deemed cell cycle regulated (7.2% of the quantified proteome).

##### Clustering of Patterns of Cell Cycle Regulation

**Fig. 3.**
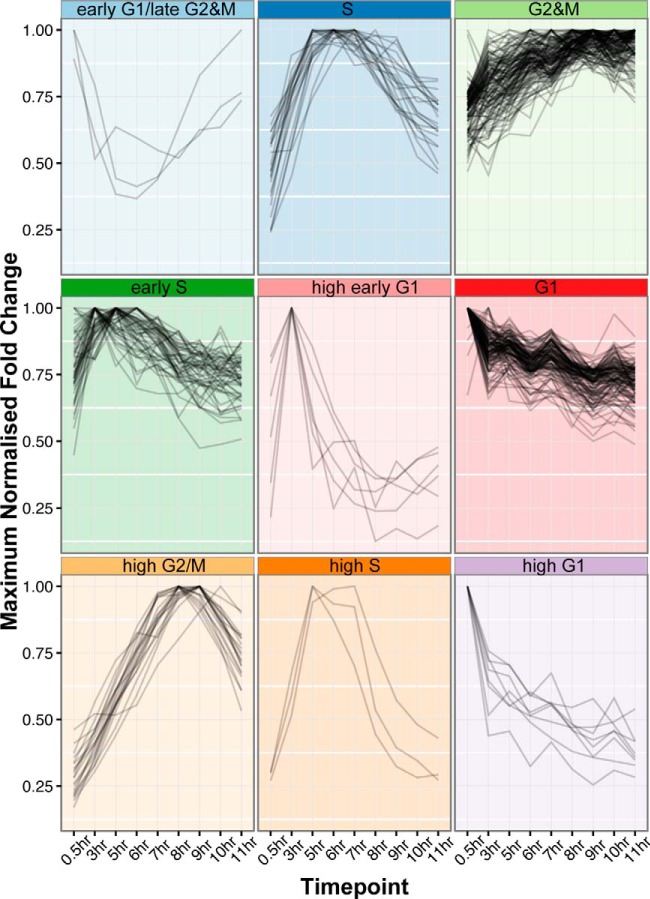
**Clustering of cell cycle regulated proteins.** Cell cycle regulated proteins were clustered into nine distinct patterns of cell cycle regulation. Clusters were named by cross-referencing the peak expression time point of each cluster to collected flow-cytometry data.

To classify proteins according to their pattern of temporal regulation, we applied the K-means clustering technique. The 384 cell cycle regulated proteins classified into 9 clusters (*n* = 9) using k-means and the fuzzy score (see Methods) (Fig. 3). Clusters were named based on the time point where peak abundance was measured and cross-referencing to the flow cytometry profiles of each time point. Clusters were classified as “high” if the mean maximum fold-change of proteins within the cluster was > 2.7. Proteins were named as “early G1/late G2 and M” (3 proteins), “G1” (129 proteins), “high early G1” (6 proteins), “high G1” (8 proteins), “S” (22 proteins), “early S” (53 proteins), “high S” (3 proteins), “G2 and M” (140 proteins) and “high G2 and M” (20 proteins) (supplemental Table S2). The gene ontology (GO) terms enriched within each cluster can be found in supplemental Table S3. The most enriched term in G1-phase clusters was “peroxisome fission,” whereas S-phase clusters were enriched for terms such as “mitochondrial DNA replication,” “DNA repair” and “DNA replication”. G2 and M-phase clusters were highly enriched for terms including “mitotic cell cycle,” “chromosome segregation,” and “kinetochore.”

**Fig. 4.**
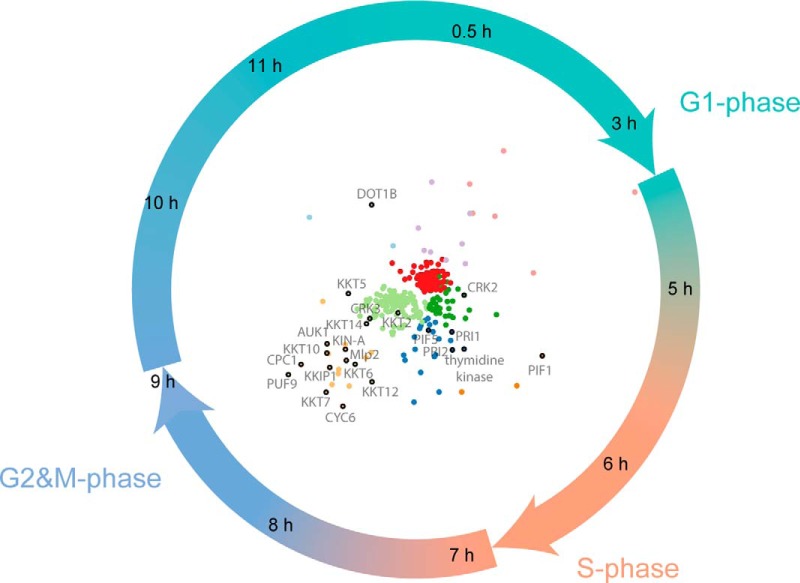
**Radial visualization plot annotated with known cell cycle regulated proteins.** Time-points are represented as individual hours on a clock-face. Individual protein groups are pulled toward the time-points they are most abundantly expressed in. Only proteins classified as cell cycle regulated are plotted with colors matching the clusters in Fig. 3. Individual proteins known to be involved in *Trypanosoma brucei* cell division are labeled.

To display the data, we have produced radial visualization plots, which is a polar coordinate system. Time-points are hours on the clock-face (*i.e.* related to the angle of the polar coordinate system) and the orthogonal axis (*i.e.* the distance) relates to the relative abundance of a protein across the time-course. A number of known cell cycle regulated proteins in *T. brucei*, such as CRK2, Mlp2, AUK1, and CPC1 are upregulated at time-points that correlate well with their described functions (Fig. 4) (4, 5, 24–27). Fifty-nine of the detected proteins were annotated with GO terms associated with the cell cycle; with fourteen of these classified as cell cycle regulated from the proteomic data set (supplemental Fig. S3). By cross-comparison to RNA interfering target sequencing (RITseq) data sets it was determined that 119 of the 384 proteins in cell cycle regulated clusters are essential for growth in one or more lifecycle stage of *T. brucei* in culture (supplemental Fig. S4) (23). Of these, 40 are annotated as hypothetical proteins of unknown function (supplemental Fig. S4). These data are also available via an open access, interactive web application (http://134.36.66.166:8883/cell_cycle).

##### Validation of Cell Cycle Regulation Through TrypTag Database

**Fig. 5.**
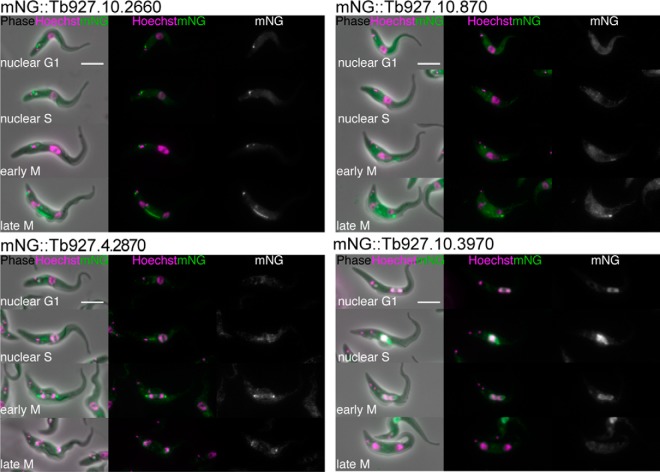
**Validation of proteomic predictions of novel cell cycle regulated proteins through TrypTag.** Selected images from TrypTag (16) high-throughput microscopy database of “hypothetical proteins of unknown function” identified as cell cycle regulated from proteomic data (*A*) mNG::Tb927.10.2660 (*B*) mNG::Tb927.10.870 (*C*) mNG::Tb927.4.2870 (*D*) mNG::Tb927.10.3970. The four panels from top to bottom displays a representative image of a cell in nuclear G1, nuclear S, early M and late M-phase of the cell cycle. Scale bar represents 5 μm. mNG, mNeonGreen.

Some of the 384 proteins classified as cell cycle regulated can be found in the TrypTag endogenous tagging database, providing microscopy images of the protein localization at different cell cycle stages as complementary evidence to evaluate cell cycle regulated patterns of expression (16). Some of these proteins are known to be involved in *T. brucei* cell division, including KIN-A, KIN13–1, Mlp2, KKT10, TOEFAZ1, FAZ18, and KKIP1 (supplemental Fig. S5) (9, 24, 25, 28–32). Furthermore, it was also possible to confirm the cell cycle regulation of four uncharacterized hypothetical proteins of unknown function (Fig. 5). Of these, three are classified into G2 and M-phase clusters (Tb927.10.2660, Tb927.10.870 and Tb927.4.2870) and the other in an S-phase cluster (Tb927.10.3970), matching the patterns of expression in cells when endogenously tagged with a fluorescent protein.

##### Comparison to Transcriptomic Data Set

We determined the overlap between proteins detected as cell cycle regulated in our proteomic data set and transcripts detected in a previously published transcriptome analysis of the cell cycle of PCF trypanosomes (supplemental Fig. S6) (10). Of the 5323 proteins quantified in this work, 93% are detected in the transcriptomic data set. Conversely 72% of the 6829 transcripts identified are matched with proteins detected in the proteomic data set. Proteomic and transcriptomic analyses classify 384 proteins and 530 transcripts, respectively, as regulated across the cell cycle, which map to a total of 836 unique genes (supplemental Table S4). In the comparison, 24 proteins and 139 transcripts in the proteomic and transcriptomic data sets, respectively, could not be compared as they were present in only one data set. Of the remaining 673 cases where direct comparison is possible, 83 are classified as regulated in both data sets. In contrast, 590 are classified as cell cycle regulated in either the proteomic data set (277), or the transcriptomic data set (313), but not both (supplemental Table S4).

GO enrichment analysis of each of these categories was performed (supplemental Table S5). Enrichment of cell cycle associated GO terms was only detected in the group of either proteins, or transcripts, identified as changing in both data sets (chromosome segregation and kinetochore), and of transcripts detected as changing only in the transcriptomic data set (DNA replication).

The 83 cell cycle regulated genes identified in common between both data sets includes CPC1, AUK1, CRK3 and multiple kinetochore proteins (KKT1, 7 and 2) (supplemental Table S4). The class of cell cycle regulated proteins whose cognate mRNA abundances were measured, but not cell cycle regulated, includes DOT1B, KIN-A, CYC4, CYC6, CRK2, and multiple kinetochore proteins (KKT5, 6, 10, 12, 14, 15 and 17) (supplemental Table S4). The set of 139 transcripts classified as cell cycle regulated, but not detected in our proteomic data set, contains CDC45, CRK10, CYC8, KIN-B, PLK and multiple kinetochore components (KKT8, 9, 11 and 13) (Supplemental Table 4). Finally, the 313 cell cycle regulated transcripts that do not show cell cycle regulation at the protein level includes components of the trypanosome flagellum and various subunits of nuclear and kinetoplastid DNA polymerases (supplemental Table S4).

A contingency table was produced to compare the cell cycle phase classification of the 83 proteins and transcripts identified as changing in both data sets (supplemental Table S6). A chi-squared test reveals that the null-hypothesis, that there is no relationship between transcript and protein classification, is false (*p* = 0.0001), indicating a positive correlation between transcript and protein cell cycle phase classification. However, we observe that transcripts peaking in abundance in G1-phase are more likely to encode for proteins that peak in abundance in S-phase (36 out of 55 transcripts), higher than would be expected for a random distribution (27 out of 55 transcripts). Furthermore, of the 55 G1 transcripts, a total of 13 peak in expression at the protein level only at G2 and M-phase. Finally, of the 21 S-phase classified transcripts, 15 are identified in G2 and M-phase clusters in the proteomic data, 87% higher than would be expected from a random distribution, whereas only 4 are classified in S-phase clusters.

##### Data Visualization

All of the processed MS data and predictions of cell cycle phase classification have been made freely available via a custom, searchable database. The data can be browsed on a web server at http://134.36.66.166:8883/cell_cycle. The web page displays an interactive radial visualization plot of the 384 proteins classified as cell cycle regulated, color coded by their cluster grouping. Clicking on individual proteins within the radial visualization plot loads their abundance profile over the proteomic time-course across three biological replicates. Plots for any of the 5325 proteins detected in the data set can also be loaded through the selection Table in the top right-hand corner of the web page. The selection Table is fully searchable, allowing input of gene ID or any term which may be associated with the gene description (*e.g.* kinase), and can be ordered by either gene ID, gene description, fold-change, Pearson correlation, or cluster classification.

## DISCUSSION

### 

#### 

##### Comparison of Elutriation Methods

The present study shows that elutriation efficiently enriches for G1-cells, (93% enrichment) and that high enrichment of S-phase and G2 and M-phase cells could be obtained by reseeding elutriated G1-phase cells. Direct enrichment of S-phase and G2 and M-phase cells by elutriation was inefficient, possibly because of limitations in resolving the size differences between S and G2 and M-phase cells. Compared with double-cut elutriation, as previously described (10), the single-cut method described in this study produced very similar enrichment efficiencies while providing a significantly higher yield of cells, which is beneficial for high proteomic coverage to capture low abundant proteins. A recently published study that thoroughly characterizes elutriation of bloodstream and procyclic form trypanosomes supports the idea that single cut elutriation is a robust, reproducible method for cell cycle phase enrichment (15).

Single-cut elutriation compares well to other methods used to produce populations enriched in different cell cycle phases. It is possible to sort cells by flow cytometry, based on DNA content, either on live, or fixed cells, for proteomic analysis (33). However, to produce ∼200–400 μg of protein per sample requires ∼1 × 10^8^ trypanosome cells, which would require very long sorting times using flow cytometry, especially for S-phase cells that constitute ∼15% of asynchronous cultures. Other methods include drug treatments to synchronize cells, such as hydroxyurea treatment (34, 35), or starvation through removal of serum from culture (36). Although drug-based synchronization methods are often more technically expedient, compared with elutriation, these methods have been shown to lead to artifactual proteome changes associated with an arrest phenotype, rather than changes that occur during a physiological, unperturbed cell cycle (37).

##### Cell Cycle Regulated Proteins

The proteomic data successfully identify proteins associated with cell division in *T. brucei*, with increases in protein expression detected at the expected time-points (Fig. 4). For example, CRK2 (a cdc2 related kinase), is upregulated at the 5 h time point, between G1 and S-phase. This is consistent with reports that CRK2 function plays a role in the G1 to S transition, as CRK2 depletion leads to a G1-phase block in *T. brucei* (4, 5). Similarly, PIF1, a DNA helicase necessary for kinetoplast DNA replication in early S-phase (38), is upregulated at the protein level between the 5 h and 6 h time-points. Thymidine kinase, necessary for genomic DNA replication (39), is upregulated between 6 h and 7 h. Furthermore, many of the proteins upregulated between 8 h and 9 h have ascribed G2 and M-phase functions, including components of the chromosomal passenger complex (AUK1, CPC1, and KIN-A) (26–28), another cdc2-related kinase (CRK3) (4), motor proteins involved in spindle assembly (Mlp2 and KIF13) (24, 25, 29, 40) and multiple kinetochore proteins (KKTs) (9). Finally, DOT1B is upregulated late in G2 and M-phase and into G1-phase. This is a histone methyltransferase known to modify chromatin as cells exit mitosis and is necessary for cell division during differentiation from bloodstream to procyclic form cells (41, 42).

##### Classification of Temporal Patterns of Protein Abundance

The 384 cell cycle regulated proteins are divided into nine clusters that we associate with four distinct cell cycle phases (G1, S, G2 and M and late G2 and M/early G1) (Fig. 3 and supplemental Table S2). The GO enrichment of individual clusters demonstrates the association of GO terms associated with expected cell cycle phases; for example, G2 and M-phase clusters are associated with GO terms such as “M-phase” and “mitotic cell cycle”, and also cellular processes associated with G2 and M phases, including “spindle assembly” and “chromosome segregation” (supplemental Table S3), supporting the idea that proteins of unknown function can be associated with roles in particular cell cycle phases based on their clustering. To this end, 46 hypothetical proteins of unknown function are observed within G1-phase clusters, 40 in S-phase clusters and 65 in G2 and M-phase clusters, indicating potential roles for these proteins in these distinct stages of cell division.

Surprisingly, 36 out of 48 proteins identified with a described cell cycle associated GO term are not classified as cell cycle regulated in our data set (supplemental Fig. S3). If a protein has a function during the cell cycle we would expect a cell cycle specific pattern of regulation, though this does not necessarily have to occur at the level of protein abundance. The proteins may, therefore, be regulated at the level of post-translational modification, or through either modification of interaction partners, or subcellular localization, whereas its abundance remains relatively constant. Another formal explanation could be that the peptides used to quantify these proteins may be suffering effects of interference, leading to ratio compression, masking real changes in protein abundance (43).

##### Comparative Analysis of the Cell Cycle Regulated Transcriptome and Proteome

Although a previously published transcriptomic analysis of the cell cycle in PCF *T. brucei* (10) identifies a similar number of genes as cell cycle regulated (530 transcripts) as identified at the level of protein (384 proteins), there is a surprisingly low overlap between these lists, with only 83 in common (supplemental Fig. S6 and supplemental Table S4).

As expected, the group of 83 proteins identified in common between both data sets contains known cell cycle regulated proteins, and the classification of this group of proteins in two independent studies increases confidence that they are genuinely cell cycle regulated (supplemental Table S4). Although there is limited overlap between the lists of proteins/transcripts identified as regulated in the proteomic and transcriptomic studies, both methodologies successfully identify known cell-cycle regulated proteins. For example, the group of 277 cell cycle regulated proteins that are not reported to be regulated at the transcript level includes several cyclin proteins, a cdc2-related kinase and seven kinetochore associated proteins (supplemental Table S4). Similarly, the 313 transcripts classified as regulated, but not corroborated at the protein level, includes proteins which may be involved in cell cycle specific functions, such as kinetoplastid and nuclear DNA replication (supplemental Table S4). The set of 139 transcripts classified as cell cycle regulated, not detected in our data set, also contains several cell cycle associated kinases, cyclins and kinetochore associated proteins (supplemental Table S4).

These results demonstrate the complementarity of both data sets, as although there is only a partial overlap in the transcripts/proteins classified as cell cycle regulated, both are successful in identifying known regulated transcripts/proteins that the other did not identify. There are several reasons why these experiments may preferentially identify different sets of transcripts/proteins. For example, utilizing proteomic techniques, it is a challenge to reliably identify and quantify low abundance proteins, as evidenced by our ability to identify only 72% of the transcripts identified. Because of restricted temporal expression, cell cycle regulated proteins may be of low abundance, hence it is no surprise that, particularly in the class of transcripts not identified in our proteomic data set, there are known cell cycle regulated proteins only identified by transcriptomics. Moreover, it is not surprising that some proteins are only identified as regulated from proteomic evidence, as protein abundance can be regulated by factors independent of mRNA abundance, such as the rates of translation and protein degradation.

There are also aspects of experimental design which may lead to the differences observed in classification of proteins or transcripts as cell cycle regulated. The proteomic study described here utilizes nine time-points compared with four in the transcriptomic study. The use of more time-points allows for a finer grained analysis of cell cycle regulation, increasing the probability of detecting proteins with significant changes within the cell cycle. Additionally, the methods for classification of a protein or transcript as cell cycle regulated are different. The proteomic data set utilizes three biological replicates of a time-course of single-cut elutriated cells, with the reproducibility and mean maximum fold-change used to classify cell cycle regulation. The transcriptomic data set uses a non-corroboration rate through the comparison of ranked fold-changes between two single replicate experiments, using either double-cut elutriation or starvation to synchronize cells in G1-phase. The lack of biological replicates makes it difficult to assess the statistical significance of the results and could lead to misassignment of cell cycle regulated transcripts (false positives). Similarly, using the comparison of ranked fold-changes of two very distinct methods of synchronization as the basis for classifying cell cycle regulation may lead to false negatives, as each synchronization procedure may have method-specific transcriptional signatures. Indeed, it is known that drug-based and elutriation-based cell cycle proteomes differ for mammalian cells (37).

Using the remaining 83 transcripts/proteins found in common to be cell cycle regulated between both data sets, we compared the classification of the cell cycle phases that the transcript and protein peaks in (supplemental Table S6). These results indicate a lag between an increase in mRNA abundance translating into an increase in protein abundance. For example, we observe that S-phase and G2 and M-phase classified proteins are mainly identified as G1 and S-phase transcripts, respectively. Alternatively, the experimental design in our proteomic study may allow for more accurate classification of peak expression, because of a higher temporal resolution, using nine time-points, compared with four in the transcriptome study.

##### Cell Cycle Regulatory Role of PSP1 Domain Proteins

We note the enrichment of polymerase suppressor 1 (PSP1) domain containing proteins within the group of 831 transcripts/proteins with evidence for cell cycle regulation. The PSP1 protein was first discovered in yeast, where it was found to suppress mutations in temperature sensitive DNA polymerases (44). The C terminus of PSP1 contains a domain that is found in up to 13 proteins in *T. brucei* (supplemental Table S6). Two of these proteins have homologs in *Crithidia fasciculata* (RBP33 and RBP45) that are subunits of the cycling sequence binding protein (CSBP II), which bind directly to mRNAs that periodically accumulate across the cell cycle. RBP33 and RBP45 are also known to be differentially phosphorylated across the cell cycle, which may regulate their interaction with mRNA (45). Of the remaining 11 PSP domain containing proteins in *T. brucei*, 4 are classified as cell cycle regulated in both transcriptomic and proteomic data sets, and one more in the transcriptomic data alone. All four proteins detected are in the top 18 most significantly changing proteins in the proteomic data, with maximum fold-changes across the cell cycle >3.6 (supplemental Table S7). As there is now evidence for cell cycle regulation of 7 out of 13 PSP1 domain containing proteins in *T. brucei*, either through changes in abundance or phosphorylation, we propose that this domain may be a conserved domain intimately involved in cell cycle associated processes in kinetoplastids.

##### Identification of Novel Cell Cycle Regulated Proteins

From the 384 proteins with patterns of cell cycle regulation, only 12 are associated with a cell cycle GO term (supplemental Fig. S3). We are therefore potentially describing novel cell cycle associated functions for hundreds of proteins in *T. brucei*. However, within this group we find a few proteins, such as PIF1, thymidine kinase and PUF9, all known to have key functions during cell division, but lacking a cell cycle-related GO annotation (38, 39, 46). This result highlights the need for better curation of trypanosomatid database resources and studies such as this can contribute evidence through the data produced. It is also clear from Fig. 4 that proteins upregulated in the G2 and M-phase of the cell cycle are more likely to be annotated, reflecting the bias in the cell cycle literature toward the study of how mitotic entry and exit is regulated.

To expand the identification of novel proteins essential for the cell cycle in trypanosomatids, our data set was filtered to only display hypothetical proteins of unknown function that are essential for the growth of the parasites in culture (supplemental Fig. S4) (23). Of the 119 essential proteins in cell cycle regulated clusters, 40 are classed as hypothetical proteins of unknown function with over 4-fold-changes across the time-course. That these proteins are changing in abundance across the cell cycle, and are essential for growth in culture, points to the idea that they are essential because of their role in cell-division. As these proteins are classed as hypothetical proteins of unknown function, lacking obvious sequence homology to proteins characterized in other eukaryotes, they could be key candidates to target with drugs because they could selectively interfere with trypanosomatid, rather than host, cell division.

##### Validation of Proteomic Data Through TrypTag

Cross-comparison of the 384 cell cycle regulated proteins to the TrypTag microscopy database, a project aiming to tag every trypanosome protein with mNeonGreen (mNG) and determine their localization, provides orthogonal evidence for the proteomic predictions of cell cycle regulation. We highlight four uncharacterised proteins, annotated as hypothetical proteins of unknown function, which show distinctive localizations during cell division (Fig. 5). Tb927.10.2660, Tb927.10.870, and Tb927.4.2870 were all found in G2 and M phase clusters from the proteomic data set, matching the patterns of localization observed by microscopy. mNG::Tb927.10.2660 exhibited a clear accumulation on the spindle during late G2 and M phase, whereas mNG::Tb927.10.870 and mNG::Tb927.4.2870 appeared on the flagellum attachment zone (FAZ) and spindle poles, respectively, similarly late in the cell cycle. mNG::Tb927.10.3970 displays a strong nuclear increase in S-phase cells, again matching the evidence from the proteomic time-course as an S-phase upregulated protein. A further seven examples are presented in supplemental Fig. S5, including three proteins initially annotated as hypothetical proteins of unknown function upon the first analysis of the data, but now characterized as TOEFAZ1, FAZ18, and KKIP1 (30–32).

In summary, this study presents the first in depth analysis of the cell cycle regulated proteome of procyclic form *Trypanosoma brucei*, identifying hundreds of cell cycle regulated proteins. This data set should be of use to the wider trypanosome research community, providing valuable functional information on uncharacterized proteins and, through the identification of essential cell cycle regulated proteins, offering a list of potential drug targets to selectively interfere with cell division in this organism. Although there is an overlap between the proteomic data and previously published transcriptomic data, there are also major differences between the two, indicating a complex relationship between mRNA and protein abundances. Finally, combining evidence from separate, large-scale proteomic data sets, such as the mass spectrometry data produced here, and the microscopy based TrypTag database, provides powerful tools to characterize protein abundance and localization of proteins in an unbiased manner.

## DATA AVAILABILITY

All mass spectrometry data have been deposited with the ProteomeXchange Consortium via the PRIDE partner repository with the data set identifier PXD008741, https://www.ebi.ac.uk/pride/archive/. Processed data and data exploration tools can be found at http://134.36.66.166:8883/cell_cycle. Annotated spectra can be viewed from the MS-Viewer website (http://msviewer.ucsf.edu/prospector/cgi-bin/msform.cgi?form=msviewer) by entering the search key, t5jurduitz.

## Supplementary Material

Supplemental Data

## Footnotes

^1^ The abbreviations used are:

CRK: Cdc2-related kinase
PCF: Procyclic form
TMT: Tandem Mass Tag
NEM: N-ethylmaleimide
TFA: Trifluoroacetic acid
PCC: Pearson correlation coefficient
MFC: Maximum fold change
GO: Gene ontology
PSP1: Polymerase suppressor 1
mNG: mNeonGreen.
