# Supplementary material for: Proteomic Analysis of the Cell Cycle of Procylic Form Trypanosoma brucei
Source: Mol Cell Proteomics. 2018 Mar 19;17(6):1184–95. doi: 10.1074/mcp.RA118.000650 (PMC5986242; doi:10.1074/mcp.RA118.000650)
Supplement: Supplemental Data [file supp_17_6_1184__index.html]

Proteomic analysis of the cell cycle of procylic form Trypanosoma brucei — Trypanosoma brucei cell cycle regulated proteome — Proteomic Analysis of the Cell Cycle of Procylic Form Trypanosoma brucei — Trypanosoma brucei Cell Cycle Regulated Proteome — Supplemental Data 

# Proteomic Analysis of the Cell Cycle of Procylic Form *Trypanosoma brucei*

## Supplemental Data

- Supplemental Information - Supplementary Figures and Tables
- Supplementary Table 2 - Processed quantitative protein groups data and prediction and classification of cell cycle regulated proteins.
- Supplementary Table 3 - Gene ontology enrichment within cell cycle regulated clusters
- Supplementary Table 4 - Comparison of transcripts and proteins classified as cell cycle regulated from transcriptomic and proteomic studies.
- Supplementary Table 5 - Gene ontology enrichment terms observed in set of genes classified as regulated in both transcriptomic and proteomic data, and set of genes classified as regulated only in transcriptomic data.
