# Supplementary material for: Proteomic Analysis of the Cell Cycle of Procylic Form Trypanosoma brucei
Source: Mol Cell Proteomics. 2018 Mar 19;17(6):1184–95. doi: 10.1074/mcp.RA118.000650 (PMC5986242; doi:10.1074/mcp.RA118.000650)
Supplement: Supplemental Data [file supp_RA118.000650_135690_1_supp_86639_p4x9gm.pdf]

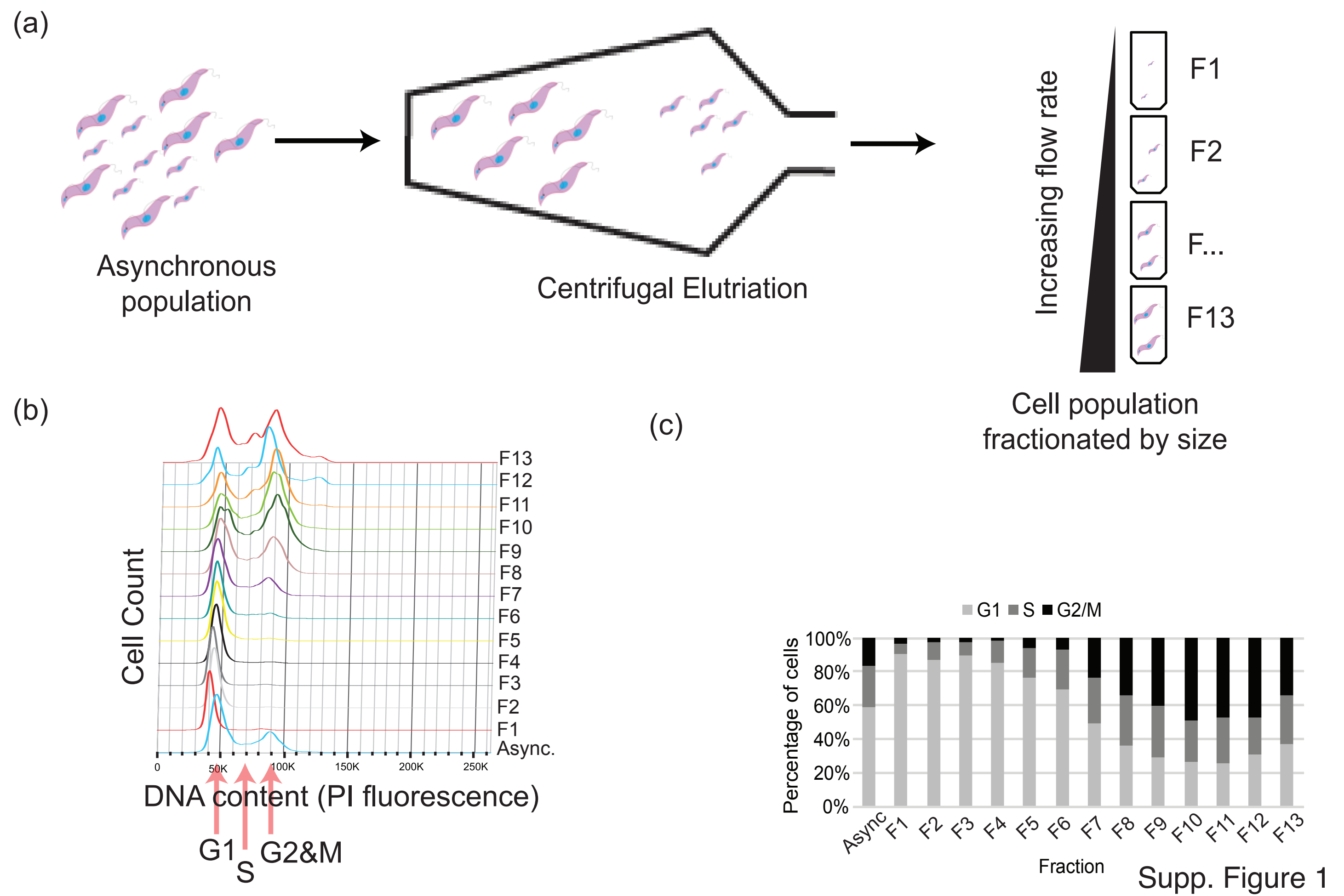

Supp. Figure 1

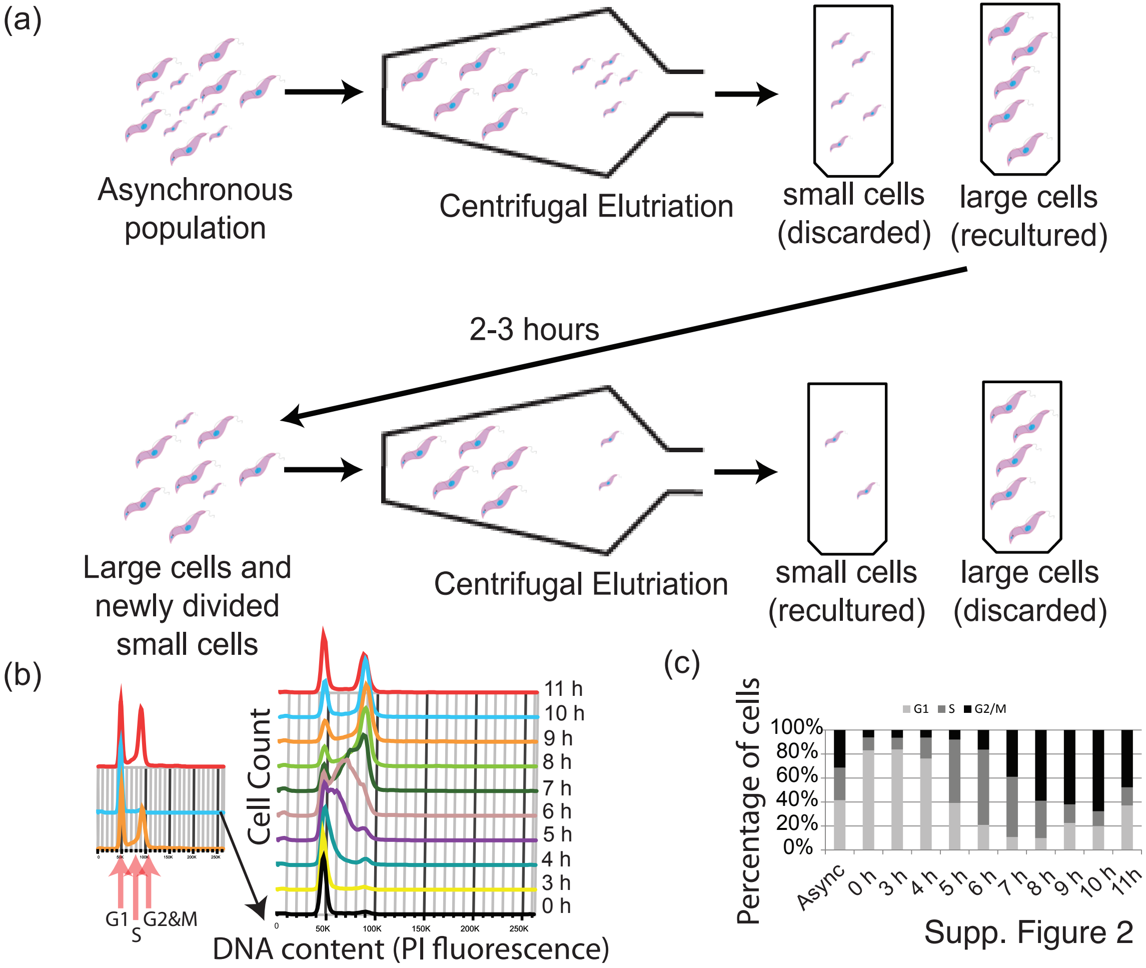

Supp. Figure 2

Supp. Figure 3

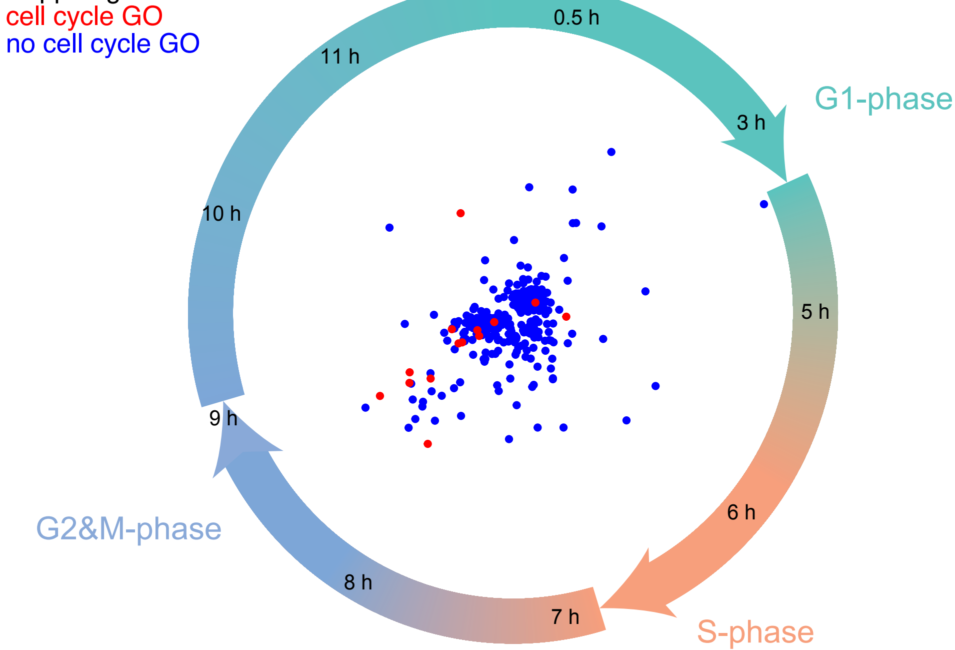

Supp. Figure 4

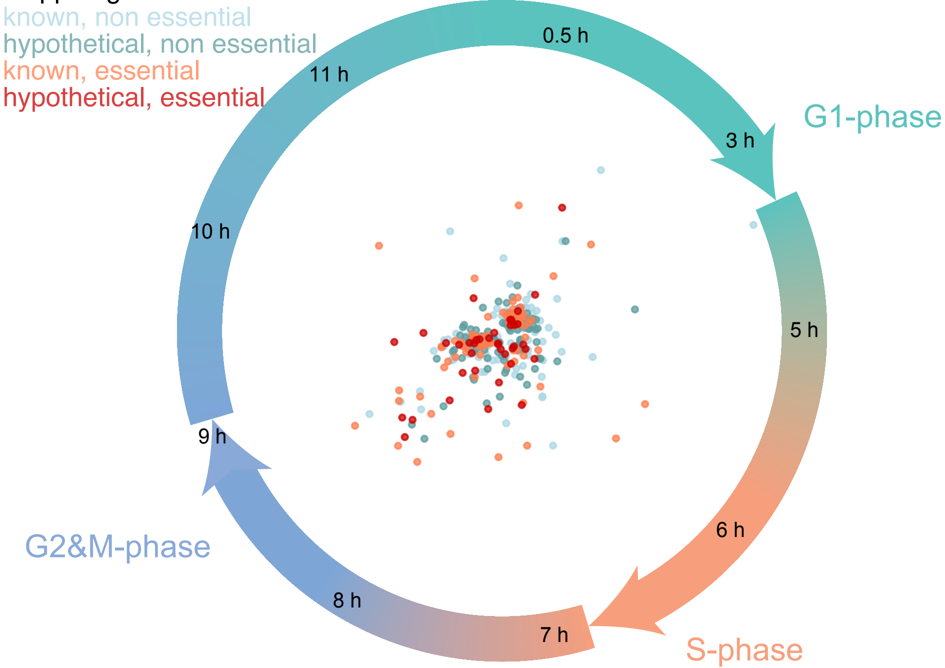

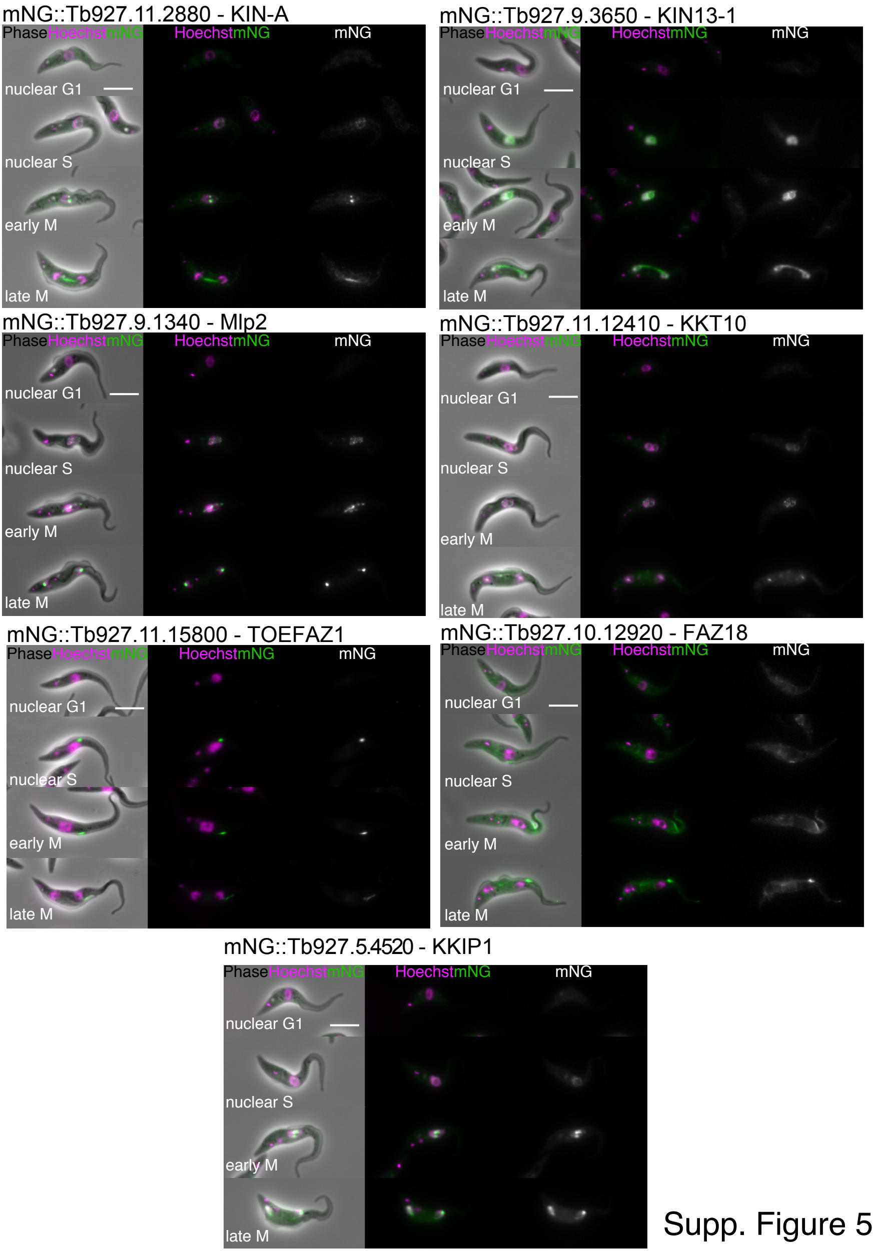

Supp. Figure 5

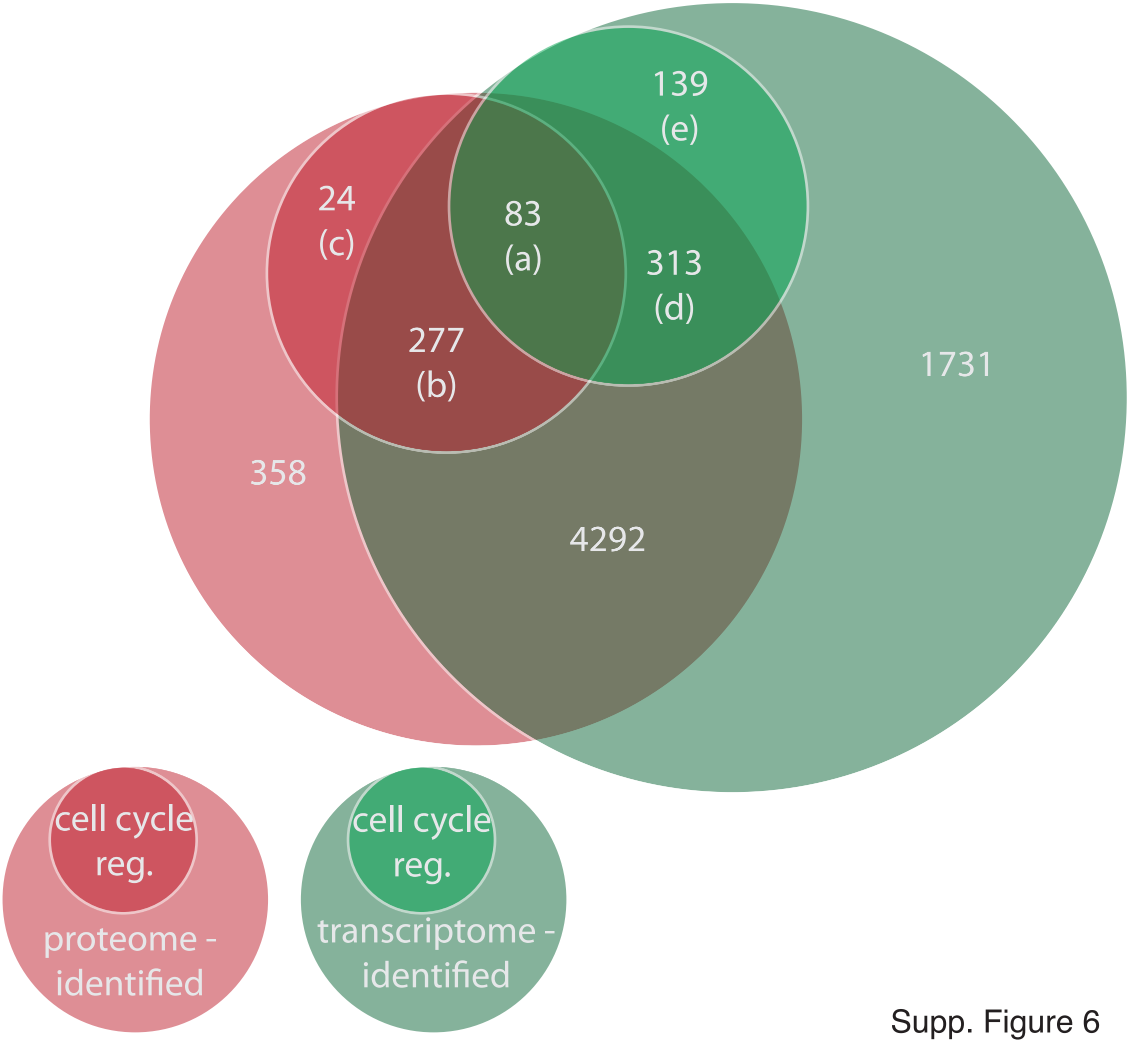

Supp. Figure 6

Supplementary Figure Captions

Supp Fig 1. Direct Elutriation.

(A) Diagrammatic representation of ‘direct’ elutriation. An asynchronous culture of procyclic form trypanosomes is loaded into the elutriation chamber and fractionated into cell populations of increasing sedimentation coefficient by gradually increasing the flow rate through the chamber (B) DNA content (PI staining) of cells harvested in distinct fractions during ‘direct’ elutriation. (C) Estimation of cell cycle distribution of cells harvested in each fraction compared to original asynchronous culture.

Supp Fig 2. Double Cut Elutriation.

(A) Diagrammatic representation of double-cut elutriation (ref). An asynchronous culture of procyclic form trypanosomes is loaded into the elutriation chamber and split into ‘small’ cells, which are discarded, and ‘large’ cells, which are recultured for 1 h. ‘Large’ cells are re-elutriated, this time keeping ‘small’ newly divided cells for the following time-course (B) DNA content (PI staining) of cells harvested during double-cut elutriation. (C) Estimation of cell cycle distribution of cells harvested from time-course compared to original asynchronous culture.

Supp Fig 3. Radial visualisation plot highlighting proteins with associated cell cycle gene ontology annotation.

Time-points are represented as individual hours on a clock-face. Individual protein groups are pulled towards the time-points they are most abundantly expressed in. Only proteins classified as cell-cycle regulated are plotted. Red proteins are associated with a cell cycle related gene ontology term.

Supp Fig 4. Radial visualisation plot categorising known/hypothetical proteins that are essential/non-essential for parasite growth in culture.

Time-points are represented as individual hours on a clock-face. Individual protein groups are pulled towards the time-points they are most abundantly expressed in. Only proteins classified as cell-cycle regulated are plotted. Blue and red dots indicate proteins classified as non-essential and essential, respectively, for at least one life-cycle stage of *T. brucei* (Alsford et al., 2011). Darker blue and red dots identify proteins with a 'hypothetical' gene name, while lighter dots identify proteins with a descriptive gene name.

Supp Fig 5. Validation of proteomic predictions of cell cycle regulation through TrypTag.

Images from TrypTag high-throughput microscopy database of proteins identified as cell-cycle regulated from proteomic data that are also classified as cell-cycle regulated from microscopy data (A) mNG::Tb927.11.2880 – KIN-A (Li et al., 2008) (B) mNG::Tb927.9.3650 – KIF13-1 (Wickstead et al., 2010) (C) mNG::Tb927.9.1340 – Mlp-2 (Holden et al., 2014; Morelle et al., 2015) (D) mNG::Tb927.11.12410/Tb927.11.12420 – KKT10/19 (Akiyoshi and Gull, 2014) (E) mNG::Tb927.11.15800 TOEFAZ1 (Sinclair-Davis et al., 2017) (F) mNG::Tb927.10.12920 – FAZ1 (Zhou et al., 2016) (G) mNG::Tb927.5.4520 – KKIP1 (D'Archivio and Wickstead, 2017). The four panels from top to bottom displays a representative image of a cell in nuclear G1, nuclear S, early M and late M-phase of the cell cycle. Scale bar represents 5 μm. mNG – mNeonGreen.

Supp Fig 6. Comparison of proteomic data to previous transcriptomic analysis (Archer et al., 2011).

Venn diagram displaying overlap between proteins and genes identified in proteomic data (red) and transcriptomic data (green). Darker shade indicate proteins or transcripts classified as cell cycle regulated in each study. (a) Identified as cell-cycle regulated in both datasets. (b) Identified in both datasets and classed as cell-cycle regulated only in proteomic analysis. (c) Identified only in proteomic data and classified as cell-cycle regulated. (d) Identified in both datasets and classed as cell-cycle regulated only in transcriptomic analysis. (e) Identified only in transcriptomic data and classified as cell-cycle regulated.

Supplementary Table 1

Cell-cycle phase	Direct	Single-cut	Double-cut
**G1**	93%	88%	83%
**S**	34%	53%	63%
**G2&M**	52%	61%	68%

Supplementary Table 6

transcriptome \ proteome	**G1**	**S**	**G2&M**	total genes
**G1**	6 *(7)*	36 *(27)*	13 *(22)*	55
**S**	2 *(3)*	4 *(10)*	15 *(8)*	21
**G2&M**	2 *(1)*	0 *(3)*	5 *(3)*	7
total genes	10	40	33	83

$\chi^2 = 22.89$, *d.f.* $= 4$, $p = 0.0001$

Supplementary Table 7

Gene ID	Gene description	Evidence for cell-cycle regulation
Tb927.3.5080	PSP1 C-terminal conserved region, putative	ID in transcriptome – not changing
Tb927.8.3850	PSP1 C-terminal conserved region, putative	ID in transcriptome – late G1
Tb927.9.9370	PSP1 C-terminal conserved region, putative	ID in transcriptome – not changing
Tb927.10.8330	*S. cerevisiae* PSP1 homologue, putative	ID in transcriptome – late G1; ID in proteome – S-phase 3.5 MaxFC
Tb927.10.9910	PSP1 C-terminal conserved region, putative	ID in transcriptome and proteome – not changing
Tb927.10.11630	PSP1 C-terminal conserved region, putative	ID in transcriptome and proteome – not changing
Tb927.11.4180	PSP1 C-terminal conserved region, putative	ID in transcriptome – late G1; ID in proteome – S-phase 3.3 MaxFC
Tb927.11.14750	PSP1 C-terminal conserved region, putative	ID in transcriptome – late G1; ID in proteome – S-phase 4.1 MaxFC
Tb927.5.760	cell-cycle sequence binding phosphoprotein (RBP33), putative	ID in transcriptome – early G1 ID in proteome – not changing Cell-cycle phosphorylation (Mittra and Ray, 2004)
Tb927.6.2000	spliceosome associated protein, putative	ID in transcriptome and proteome – not changing
Tb927.6.2850	ESAG associated protein, putative (PIE8)	D in transcriptome – S; ID in proteome – S 4.3 MaxFC
Tb927.10.9330	hypothetical protein, conserved	ID in transcriptome and proteome – not changing
Tb927.11.7140	cell-cycle sequence binding phosphoprotein (RBP45), putative	ID in transcriptome and proteome – not changing Cell-cycle phosphorylation (Mittra and Ray, 2004)

Supplementary Table Captions

Supp Table 1. Comparison of maximum cell cycle enrichment using different elutriation methodologies.

Maximum enrichments achieved for each cell cycle population using either direct, single-cut or double-cut elutriation, as measured by flow cytometry.

Supp Table 2. Quantitative proteomic results and data analysis.

Table containing all genes identified in proteomic analysis, together with calculated maximum fold-change, classification of cell cycle regulation, cluster classification and median normalised TMT intensities for each time-point.

Supp Table 3. Gene ontology enrichment within cell cycle regulated clusters

Supp Table 4. Comparison of proteins and transcripts classified as cell cycle regulated.

Table of any gene classified as cell cycle regulated in proteomic or transcriptomic dataset. Genes are categorised by whether they were identified and cell cycle regulated in each dataset. Genes classified as regulated display the cell cycle phase classification from the appropriate dataset, along with the protein group ID from Supp. Table 2 if detected at the protein level.

Supp Table 5. Gene ontology enrichment of proteins/transcripts listed in Supplementary Table 4.

Supp Table 6. Comparison of cell cycle phase classification of proteins/transcripts identified as cell cycle regulated at protein and transcript level.

Contingency table comparing the cell cycle phase classification of proteins/transcripts identified as cell cycle regulated in proteomic and transcriptomic datasets. Numbers in black, show the observed values, and numbers in brackets in grey, show the expected values if the null hypothesis were true, with no relationship between protein and transcript classification.

Supp Table 7. Evidence of cell cycle regulation for PSP1 C-terminal domain containing proteins in *T. brucei*.

Supplementary References

Akiyoshi, B., and Gull, K. (2014). Discovery of unconventional kinetochores in kinetoplastids. Cell *156*, 1247–1258.

Alsford, S., Turner, D.J., Obado, S.O., Sanchez-Flores, A., Glover, L., Berriman, M., Hertz-Fowler, C., and Horn, D. (2011). High-throughput phenotyping using parallel sequencing of RNA interference targets in the African trypanosome. Genome Research *21*, 915–924.

Archer, S.K., Inchaustegui, D., Queiroz, R., and Clayton, C. (2011). The cell cycle regulated transcriptome of Trypanosoma brucei. PLoS ONE *6*, e18425.

D'Archivio, S., and Wickstead, B. (2017). Trypanosome outer kinetochore proteins suggest conservation of chromosome segregation machinery across eukaryotes. The Journal of Cell Biology *216*, 379–391.

Holden, J.M., Koreny, L., Obado, S., Ratushny, A.V., Chen, W.-M., Chiang, J.-H., Kelly, S., Chait, B.T., Aitchison, J.D., Rout, M.P., et al. (2014). Nuclear pore complex evolution: a trypanosome Mlp analogue functions in chromosomal segregation but lacks transcriptional barrier activity. Molecular Biology of the Cell *25*, 1421–1436.

Li, Z., Umeyama, T., and Wang, C.C. (2008). The chromosomal passenger complex and a mitotic kinesin interact with the Tousled-like kinase in trypanosomes to regulate mitosis and cytokinesis. PLoS ONE *3*, e3814.

Mittra, B., and Ray, D.S. (2004). Presence of a poly(A) binding protein and two proteins with cell cycle-dependent phosphorylation in Crithidia fasciculata mRNA cycling sequence binding protein II. Eukaryotic Cell *3*, 1185–1197.

Morelle, C., Sterkers, Y., Crobu, L., MBang-Benet, D.-E., Kuk, N., Portalès, P., Bastien, P., Pagès, M., and Lachaud, L. (2015). The nucleoporin Mlp2 is involved in chromosomal distribution during mitosis in trypanosomatids. Nucleic Acids Research *43*, 4013–4027.

Sinclair-Davis, A.N., McAllaster, M.R., and de Graffenried, C.L. (2017). Functional analysis of TOEFAZ1 uncovers protein domains essential for cytokinesis in Trypanosoma brucei. J. Cell. Sci. jcs.207209.

Wickstead, B., Carrington, J.T., Gluenz, E., and Gull, K. (2010). The expanded Kinesin-13 repertoire of trypanosomes contains only one mitotic Kinesin indicating multiple extra-nuclear roles. PLoS ONE *5*, e15020.

Zhou, Q., Hu, H., and Li, Z. (2016). An EF-hand-containing Protein in Trypanosoma brucei Regulates Cytokinesis Initiation by Maintaining the Stability of the Cytokinesis Initiation Factor CIF1. Journal of Biological Chemistry *291*, 14395–14409.
